# Guarding Embryo Development of Zebrafish by Shell Engineering: A Strategy to Shield Life from Ozone Depletion

**DOI:** 10.1371/journal.pone.0009963

**Published:** 2010-04-01

**Authors:** Ben Wang, Peng Liu, Yanyan Tang, Haihua Pan, Xurong Xu, Ruikang Tang

**Affiliations:** 1 Center for Biomaterials and Biopathways, Zhejiang University, Hangzhou, Zhejiang, People's Republic of China; 2 Wyss Institute for Biologically Inspired Engineering, Harvard University, Boston, Massachusetts, United States of America; 3 State Key Laboratory of Silicon Materials, Zhejiang University, Hangzhou, Zhejiang, People's Republic of China; University of Birmingham, United Kingdom

## Abstract

**Background:**

The reduced concentration of stratospheric ozone results in an increased flux of biologically damaging mid-ultraviolet radiation (UVB, 280 to 320 nm) reaching earth surfaces. Environmentally relevant levels of UVB negatively impact various natural populations of marine organisms, which is ascribed to suppressed embryonic development by increased radiation.

**Methodology/Principal Findings:**

Inspired by strategies in the living systems generated by evolution, we induce an extra UVB-adsorbed coat on the chorion (eggshell surrounding embryo) of zebrafish, during the blastula period. Short and long UV exposure experiments show that the artificial mineral-shell reduces the UV radiation effectively and the enclosed embryos become more robust. In contrast, the uncoated embryos cannot survive under the enhanced UVB condition.

**Conclusions:**

We suggest that an engineered shell of functional materials onto biological units can be developed as a strategy to shield lives to counteract negative changes of global environment, or to provide extra protection for the living units in biological research.

## Introduction

The global environment keeps on changing dramatically by anthropogenic activities and one of the most serious problems is stratospheric ozone depletion [1]. For nearly a billion years, the ozone layer in the atmosphere has guarded the organisms on Earth from the lethal doses of UVB [2]. Currently, 50% reduction of O_3_ concentration has been observed over Antarctica during the austral spring and the ozone hole has been world-widely documented [3], [4]. As a result, more UVB radiation reaches the earth's surface than ever before and living organisms are critically challenged to the rigors of the biologically damaging ray [5]. It has been confirmed that the exposure to UVB decreases phytoplankton productivity [6] and damages various aquatic larvae [7], [8]. For example, it leads to the development inhibition and brain lesions of anchovy; ∼50% of the anchovy cannot survive at a cumulative DNA effective dose of 0.115 J/cm^2^ over a 4-day period [7]. The developmental activity and minimal morphological complexity of embryos and larvae make them more vulnerable than adult animals [9]. Accumulated ambient UVB exposure during early stages of development can cause developmental delays and/or lethality that will affect recruitment to adult populations [10], [11], [12].

In the natural world, biological systems can use nanometer-scale architectures to produce exceptional optical effects. There exists a variety of natural photonic structures such as a species of brittlestar, *Ophiocoma wendtii*, uses photonic elements composed of calcite to collect light [13]. As well as enhancing transparency in certain insects, optical scattering from specific tapered nanostructures on and within each scale features significantly increases incident light absorption by the pigment and subsequently enhances the visual appearance of the blackness [14]. Coccolithophores are unicellular algae distinguished by a covering of calcium carbonate plates, called *coccoliths*. It has been reported that the crystalline photonic structure of certain *holococcoliths* enhances UV backscattering and can exert a protective effect by reflecting UV light. This implies that an ecological advantage represents an evolutive adaptation of some holococcolithophores [15]. Another example, egg shell is an adaptation to protect a single-cell with mineral coating. Natural biological structures are providing inspiration for technological strategy.

Inspired by these strategies in nature, fabricating a single fertilized egg with a suitable coat may make it more robust in harsh environments. It can be designed to shield living organisms from UV or other environmental invasions. It is known that special *coccoliths* have a periodic structure of calcite crystallites and form the cell covering which can filters radiation wavelengths below 400 nm more efficiently and has great total amount of radiation backscattered [15]. Similarly, we can make use of UV-absorbable matter, such as rare earth materials lanthanide phosphate (LnPO_4_) [16], as the components of cell coating to decrease UV penetration. The previous studies have concluded that the suppress effect of UVB on hatching success is greater in fish specimens than in others [12]. In this report, the eggs of zebrafish, *Danio rerio*, are used as a model to demonstrate the protected embryonic development under increased UVB radiation. Zebrafish is an important experimental vertebrate in biology because mutations induced by chemicals, radiation, or viral insertion cause visible changes (phenotypes) that can be readily observed in this vertebrate [17].

## Results

### Shell engineering

**Figure 1:**
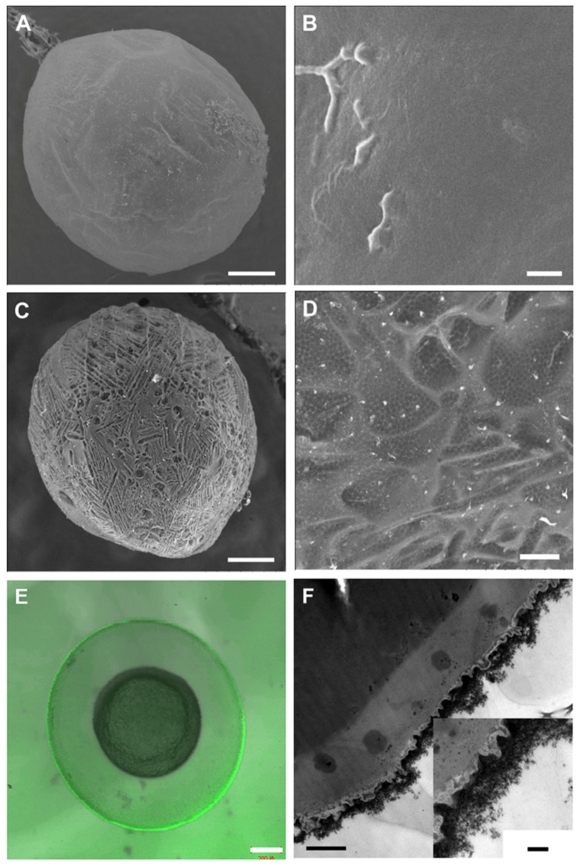
Images of the bare embryo and the coated embryo. **A.** SEM images of the bare embryo and (**B**) surface of the bare embryo. **C.** SEM images of the coated embryo with mineralized shell and (**D**) porous surface of the shell. **E.** Laser scanning microscopy image of the coated embryo with green-emission fluorescent shell. **F.** Transmission electron microscopy (TEM) the cross section image of the shell-engineered embryo. Scale bars, 200 µm (**A, C, E**), 2 µm (**B, D**), 1µm (**F**), 200 nm (**F**, the insert figure).

**Figure 2:**
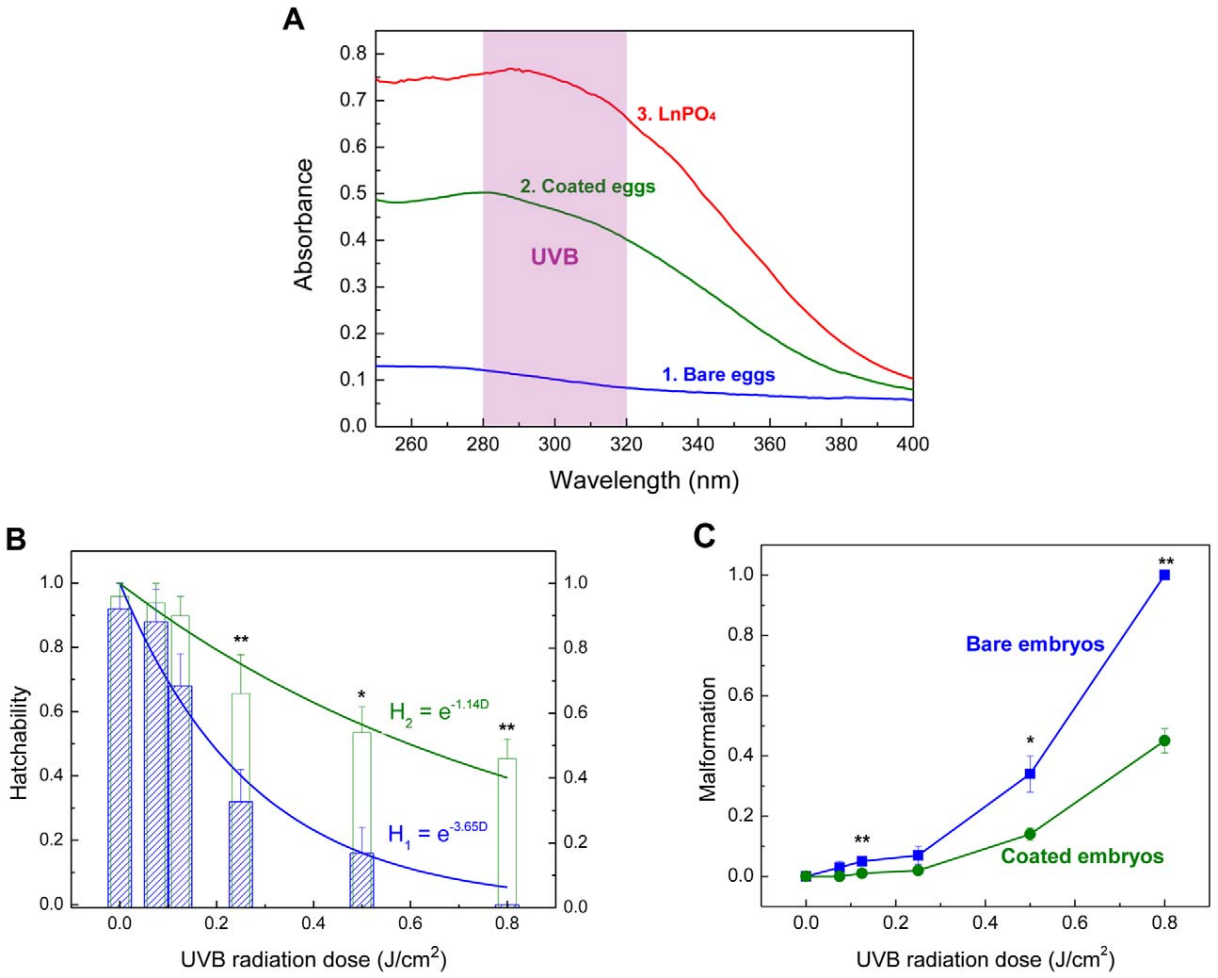
Absorption spectra and the shielding function of the shell. **A.** Absorption spectra of bare embryo, coated embryo within LnPO_4_ shell, and LnPO_4_ particles in water (2.4 g/L). **B.** Hatchability of the bare embryos and the coated ones under different dosage of UVB radiation. (R_1_^2^ = 0.96; R_2_^2^ = 0.83). **C.** Deformity percentage of embryos under different UVB doses in a short-term exposure. (* p<0.05; ** p<0.01 by Student's t-test, mean ± s.d., n = 8).

In theblastula period, the natural eggs of zebrafish are not covered by any mineral layer (Figs. 1A and 1B) and their surfaces are relatively smooth under scanning electron microscopy (SEM). Four major polypeptides (116, 97, 50 and 43 kDa) and several minor bands have been revealed reproducibly in the isolated and purified chorions of the cell membranes. Both the 116 kDa and 50 kDa polypeptides are N-linked glycoproteins. It has been shown that the 97 kDa and 43 kDa polypeptides are not glycosylated [18]. Mineral cannot be fabricated onto the eggs surface directly due to the lack of “mineralization-related factors” on their interface. It is generally agreed that the substrates containing regions rich in carboxylates or other charged functional groups are most active in the mediation of biomineralization [19], [20]. Based on those, we propose a method to enhance the mineralization skill of living cells by using layer-by-layer (LbL) technique [21]. The carboxylate-rich macromolecules, poly (acrylic sodium) (PAA), can be adsorbed onto the yolk-sac to induce a biomimetic mineralization (Text S1). The natural eggshell is thick (at a scale of millimeter) with main inorganic composition of calcium carbonate [22]. The man-made shell is ultra-thin (at a scale of hundreds nanometer) to ensure cell viability. A thin calcium layer cannot block UVB penetration, but we note that rare earth materials can absorb UVB effectively [16]. A lanthanide phosphate (LnPO_4_) coat is thereby designed for the embryos. The selected chemical composition is La_0.20_Ce_0.45_Tb_0.35_PO_4_, which is an optimal structure for UVB adsorption (Figure 2A).

After induced *in situ* mineralization, the zebrafish embryos are fully enclosed by the mineral phase. In the blastula period, zebrafish embryos can be fully coated by the mineral phase after the biomimetic mineralization (Figures 1C and 1D). The deposition of LnPO_4_ onto the chorion is confirmed by electron energy dispersive X-ray spectroscopy (Figure S1). It is mentioned that the LnPO_4_ photonic solid can turn UV into green-lights so that the shell fluorescence can be observed under UV rays [16]. The egg-like embryo-shell structure is also confirmed by confocal laser scanning microscope (Figure 1E). Excited by UV light, the green shell implies that the embryo is surrounded by LnPO_4_ (Figure S2). The mineral layers are porous so that the environmental molecules such as H_2_O and O_2_ can be transported to the enclosed cells readily. The typical thickness of the shell is about 500 nm (Figure 1F). For engineered shell modification, biocompatibility is an important parameter and should be considered. It was identified that these lanthanide elements had relatively low animal toxicities. The LD_50_ (p.o.) values (lethal dose fifty per oral route, calculated for a substance orally adsorbed causing the death of 50% of an animal population) of La(NO_3_)_3_, Ce(NO_3_)_3_, Tb(NO_3_)_3_ are 4500, 4200, and >5000 mg/kg, respectively [23]. Our specific toxicity test results also show that the LD_50_ values of La^3+^, Ce^3+^, Tb^3+^ for zebrafish embryos are 1.93±0.03, 1.10±0.11, 1.86±0.05 mM respectively (Text S1). The viabilities of the embryos after the LbL treatment and the shell engineering are 92±6% and 84±7%, respectively (Figure S3). In this examination, the viability of enclosed embryos can be estimated by their appearances easily. The dead embryos turn white since their yolks are blurry. However, the viable ones are still transparent and bright. These transparent embryos can develop into larvae successfully. However, none of the whitened embryos can develop into larvae, implying that they are dead. Thus, the egg appearances can be used as an easy but effective index of the viability. It should be mentioned that only the viable eggs (transparent ones) are collected for the subsequent UV exposure tests.

### Spectrum

The UVB shielding effect of the engineered embryos is examined. There is no significant adsorption for the bare embryos in the UV range of 280–400 nm (curve 1, Figure 2A), implying transparent feature. Thus, the inner structure of the embryo such as yolk and DNA are exposed to the penetrating UVB radiation. A rounded hump in this curve was attributed to proteins (280 nm) and nucleic acid (260∼265 nm) within the biosystem [24]. However, a strong adsorption band in the UVB range is detected for the enclosed embryos (curve 2, Figure 2A). Furthermore, the effective adsorption in UVC band (200–280 nm) and partially adsorption of UVA (320–400 nm) are also shown. These adsorption features are similar with those of pure LnPO_4_ particles (2.4 g/L) in solution (curve 3, Figure 2A). The functional LnPO_4_ shell can absorb UV effectively and around 50% penetrations of UVB rays can be blocked. Thus, the enclosed embryos receive much less UVB. Since the man-made mineral layer is ultra-thin, the transparency of the embryos under the visible lights is not influenced.

### Short-term exposure

To confirm the LnPO_4_ shells protection of embryonic development, a microprocessor controlled UVB irradiation system, Bio-Sun (Vilber Lourmat, France), is used to simulate the solar UVB emission. Cells can be inactivated and lose their reproduce ability by an exposure to UVB [24]. Inactivation is a stochastic process and is normally characterized by survival curves, a plot of the fraction of survivors as a function of UVB dose. The hatchability of embryo, *H*, decreases by a constant fractional amount for a given increment in exposure, which can be expressed by,
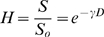
(1)where *S* and *So* are the survived (hatched) and initial amounts of the embryos, respectively, *D* is UVB dose. *γ* is a factor that characterizes biological sensitivity, lower value implies less sensitive (or stronger) of the species under UVB. Irradiation levels are based upon predicted UVB increases that will result from anthropogenic diminution of earth's protective ozone layer [6], [7]. Bio-Sun can control the UVB energy precisely. Each experimental run, 50 embryos are exposed to a preset dose (within 6 min) in Bio-Sun and then they are transferred into 10 ml egg water at 28.5°C. The hatchability is examined after 4 days. The data calculated are from at least 10 independent runs. In the control experiment without UV exposure, almost bare zebrafish embryos (92±8%) can be hatched into the early larvae. For the enclosed embryos with the LnPO_4_ shell, about 95% of them can develop into larvae successfully without UV exposure, and no abnormal phenotype is observed during the development (Figure S3). These two results show that the artificial mineral layers do not have any negative effect on the embryo developments of zebrafish. Under a relatively low UVB dose (*D*) of 0.075 J/cm^2^, *H* of the bare ones keeps at 88±10%. But the further increasing of UVB dose results in a dramatic decreasing of *H* of the bare embryos. At *D* of 0.25 J/cm^2^, only 32±10% bare embryos can evolve into early larvae. When the UVB irradiation dose increased to 0.50 J/cm^2^, the bare embryos exhibited significant deformity, 34±6% (Assessment criteria was provided in Text S2). Further increasing of *D* to 0.80 J/cm^2^ leads to a complete death of the bare embryos before hatch, which was considered as 100% malformation (Figure 2C). This tendency is in agreement with the natural phenomenon of larva population drop under ozone hole [12]. The relationship between *H* and *D* is in good agreement with equation 2 (Figure 2B). The estimated *γ* of the bare embryos under UVB is 3.65 and LD_50_ is 0.19 J/cm^2^. However, the LnPO_4_ coated embryos can receive an extra protection. At UVB doses of 0.25 J/cm^2^ and 0.80 J/cm^2^, 66±12% and 46±6% of them remain viable and then develop to the early larvae, respectively. Also, the percentages of malformation for the coated embryos are much lower than the bare ones. When UVB doses at 0.25 and 0.80 J/cm^2^, the percent deformities of coated embryos are only 2±1% and 45±4%, compared with 7±3% and 100% for the bare ones, respectively (Figure 2C). Even at low *D*, *H* of the engineered embryos is also higher than the bare ones. After the shell engineering, the value of *γ* is reduced to 1.14, indicating that the enclosed embryos are more robust under UVB. Accordingly, LD_50_ is increased to 0.54 J/cm^2^ by the coating treatment.

### Long-term exposure

**Figure 3:**
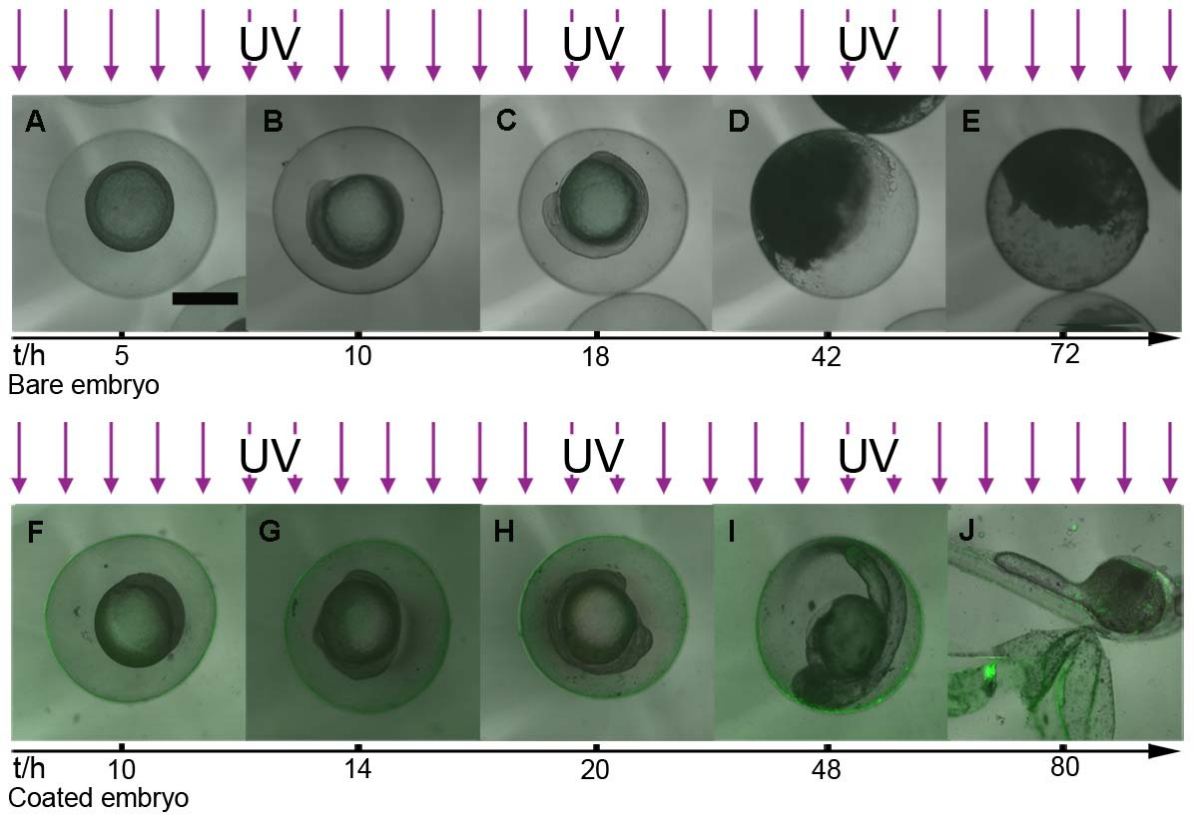
Parallel development stages of the bare embryo and the coated embryo under UV radiation. **A–E.** The development traces of the bare embryo under UVB ray. **F–J.** The development stages of the coated embryo under UV radiation. **A, F.** 30%-epiboly stage. **B, G.** Bud stage. **C, H.** 14-somite stage. **D, E.** Interruption of the development. **I.** High pectoral stage. **J.** Protruding-mouth stage. Embryonic development within engineering shell under UVB radiation is fulfilled and the man-made shell can be broken by the larva. Scale bar, 500 µm.

**Figure 4:**
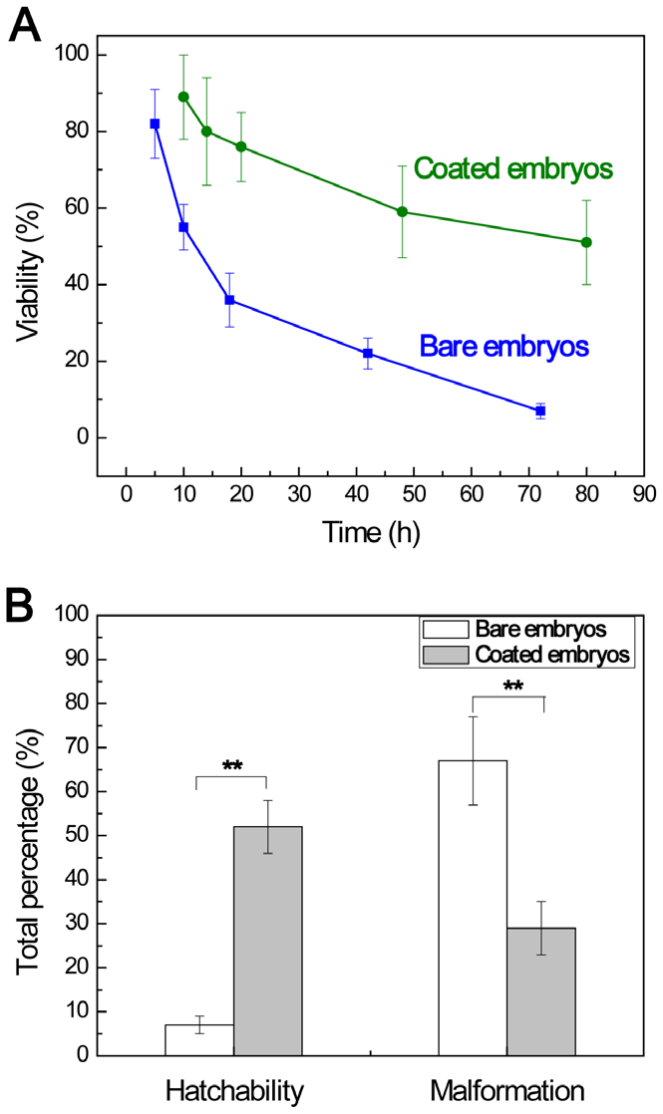
Comparison of the bare embryo and the coated ones under long-term UV exposure. **A.** Viabilities of embryos at different stages during the long-term exposure experiments. **B.** Hatchability (%) and malformation (%) of bare embryos and coated embryos in the long-term exposure (** p<0.01 by Student's t-test, mean ± s.d., n = 8).

The morphogenesis developments of zebrafish within LnPO_4_ shells under continuous UVB radiations are also examined. A sunshine simulator is used to simulate the UVB in sunshine with an intensity of 4.70 µW/cm^2^. The UVB dose in one light/dark cycle (14 h/10 h) is 0.24 J/cm^2^. Seven broad periods are usually defined for the development of zebrafish embryo: the zygote (0–0.75 h), cleavage (0.75–2.25 h), blastula (2.25–5.25 h), gastrula (5.25–10 h), segmentation (10–24 h), pharyngula (24–48 h), and hatching (48–72 h) periods [25]. Large scale death of bare embryos (∼86%) can be observed at the pharyngula stage (Figure 3) with no more development. Within ∼24 h, half of the bare embryos are opaque and albescent, indicating the lethality and failure of hatchability, which agrees with the results of the Bio-Sun study (Figure 2B). With the accumulation of UVB dose, more and more bare embryos are dead. Eventually, after accumulated irradiation dosage 0.71 J/cm^2^, 7±2% of the bare embryos can survive in 3 days. However, more than half of the enclosed embryos (51±11%) can develop favorably under the same condition (Figure 4A). At the end of experiments, the larvae can burst the shell to complete the morphogenesis development (Figure 3J). The featured green light surrounding the embryos indicates the presence of UVB radiation and LnPO_4_ shell during the experiment. However, it is noted that the embryogenesis time period of the enclosed embryos is elongated to 80 h. In comparison, the typical development period of zebrafish is 72 h [25]. The prolonged period may be explained by that the shell establishes a new niche for the enclosed embryos. The trend of hatchability and malformation between bare embryos and the enclosed group during long-term exposure are in good agreement with the results obtained by the short-term tests (Figure 4B).

## Discussion

**Figure 5:**
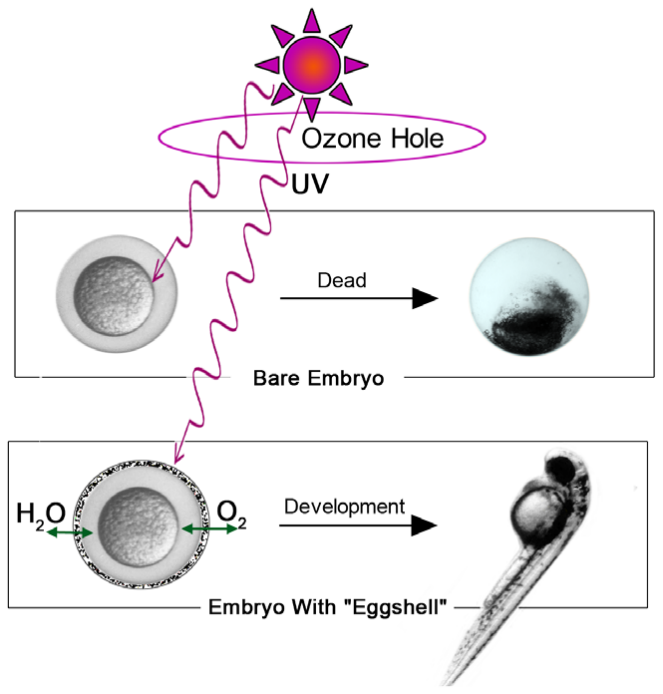
Schematic illustration of the protection by shell engineering. The coated embryo with mineral shell can develop and hatch successfully within the man-made shield under ozone hole but the bare one cannot accomplish the embryonic development.

Herein we demonstrate a strategy to protect embryo and embryonic development in the presence of UVB radiations using an artificial shield (Figure 5). The current work developed a method to fabricate functional shells for animal cells, and illustrating vital meaning of shell engineering for protection of cell or organism. This method can be developed to guarding the cells or organisms under the harmful conditions to counteract the negative change of global environment or other aggressions. For example, Ozone depletion has been most dramatic over Antarctica, where ozone levels typically decline >50% during the austral spring ‘ozone hole’ [3], [4], [12]. In our systems, the ultra-thin LnPO_4_ shell can absorb UV effectively and around 50% penetrations of UVB rays can be blocked. It is demonstrated that an engineered ultra-thin mineral coat with functional materials can shelter living lives from increased UVB flux on the earth surface effectively. Furthermore, such a coated treatment can be developed into a broad methodology in biological engineering research to provide extra protections for living organisms. For example, it is acknowledged that the viabilities of cells encapsulated in the photocrosslinking hydrogel are affected seriously by UV radiation [26]. However, the method developed in this report may be a possible approach to decrease cell damage caused by UV radiation. Also, the variety of micro- and nano-fabrication, different functional materials can offer a tool kit for cell or organism modifications and protections.

The viabilities of the embryos after shell engineering (Figure S3) imply biocompatibility of these biomimetic modifications. However, we still suggested that the toxicities could be further reduced by an optimal combination of LbL assembly and biomimetic mineralization. For instance, we could modify the surfaces of embryos by using other biocompatible polyelectrolytes. We can shelter the organisms from UVB radiation not only by UV-adsorption materials, but a crystalline photonic structure which enhances UV backscattering and could also exert a protective effect by reflecting UV light.

Thanks to its transparency, the zebrafish embryo facilitates analysis of mutations because changes in its phenotype can be tracked at the level of the individual cell in the living animal. Mutations induced by chemicals, radiation, or viral insertion cause visible changes that can be readily observed in this vertebrate [17]. It shows that the LnPO_4_ coat guards the embryos under UVB radiation with less morphology mutation. A further study of the molecular genetics about the protected zebrafish is still necessary to understand the influences of shell engineering on embryonic development comprehensively.

Although the shell engineering is achieved by a chemical method in this study, we suggest that an integration of coating technology with genetic engineering could endow the living cells with the ability of synthesis spontaneously the functional shells to encapsulate themselves. It can be achieved by incorporating biomineralization-related genes into the genome of target-cells, which can be inherited by offspring. Such an “artificial evolution” of living organisms is envisaged to hatch out “supercells” in the near future.

## Materials and Methods

### Shell engineering

Fertilized eggs were obtained from natural mating of adult zebrafish (wild type; Tübingen) from Laboratory of Molecular Genetics and Development at Zhejiang University. Adult fish were maintained at 28.5°C with a lighting schedule of 14 h light and 10 h dark, which was prepared according to *The Zebrafish Book* [27]. Eggs were collected within 1 hour post fertilization (hpf), rinsed, and placed into a clean petri dish. 0.5 g/L of chitosan (pH = 6.76, low molecular weight, Aldrich) and poly (acrylic sodium) (PAA, MW = 5,100 Da, Fluka) in embryo medium (Table S1) were used. Three-cycles, (Chitosan/PAA)_3_, polyelectrolyte-coated eggs were obtained by the LbL technique (Text S1). Appropriate amounts of La_2_O_3_, Ce(NO_3_)_3_•6H_2_O, and Tb(NO_3_)_3_•5H_2_O were dissolved in 1.0 M HNO_3_ aqueous solution. Then the solution was diluted into 50 ml 1 mM Ln(NO_3_)_3_ solution (Table S1), which contained 0.2 mM La^3+^, 0.45 mM Ce^3+^, 0.35 mM Tb^3+^. The LbL-treated eggs were put into the Ln solution and titrated with 50 ml 1mM Na_2_HPO_4_ solution (Table S1) at a rate of 1 ml min^−1^. During the reaction, pH of the reaction solution was maintained at 7.2 by a pH-constant meter (842 Titrando, Metrohm, Swltzerland) using 0.01M NaOH solution.

### Characterization

The lyophilization bottles with the bare or enclosed embryos were put into a board of −70°C of lyophilizer (Advantage EL, Virtis, USA). The procedure of the lyophilization was −60, −50, −40, −30°C for 3 h, and −20, −10, 0, 10°C for 2 h. The vacuum was 13.3 Pa. The specimens were conserved in 4°C refrigerator after the lyophilization. They were characterized by scan electron microscopy (TM-1000, Hitachi, Japan & SIRION-100 field-emission scanning electron microscope, FEI, Holland). Biological TEM was performed using JEM-1230EX (JEOL, Japan). The specimens were fixed with glutaraldehyde, OsO_4_, and K_2_Cr_2_O_7_, and were dehydrated in ethanol/acetone. They were embedded in an Epon 812/Araldite M resin. Thin sections (80±10 nm) were cut by a Reichert ultratome (Zeiss, Germany) and were stained with uranyl acetate and lead citrate. Fluorescence study was carried out by confocal laser scanning microscope (LSM 510 META, Carl Zeiss, Germany).

### Spectrum

The bare embryos and enclosed ones were packed homogenously in pure water and the absorption spectra were taken on a UV-2550 spectrophotometer (Shimadzu, Japan).

### Short term exposure

The specimens were subjected to UVB irradiation in Bio-Sun system (Vilber Lourmat, Marne-la-Vallée, France) in petri dishes (diameter of 9 cm) and the radiation wavelength was 312 nm. The doses were set in the range of 0.025–0.8 J/cm^2^ (Table S2). The specific radiation was emitted from a 30-W fluorescent tube (T-20M) above the sample tray and was automatically adjusted 4 times per second by a microprocessor with an error control of less than 1 µJ/cm^2^. After mineralization treatment, 50 embryos (in the gastrula stage) with 5 ml egg water (Text S3) in a sterile petri dish were exposed to a preset dose of UVB radiation 0.025, 0.075, 0.125, 0.25, 0.5, and 0.8 J/cm^2^ (within 6 min) in Bio-Sun successively and then they were transferred into 20 ml egg water. During the incubation, the lighting cycle was 14 h light and 10 h dark (without UVB). After 3 days, the surviving (hatching successfully) larvae were quantified. At least ten independent experiments were performed to investigate the hatchability. The bare embryos were operated in the same procedure. The experiments were carried out at temperature of 28.5°C. The statistical analyses were performed using SPSS 16.0 (©SPSS Inc. 1989–2007).

### Long-term exposure

Outdoor radiation conditions were simulated with a sunshine simulator (Text S4). A 400-W discharging lamp (MSR 400 HR, Philips, Netherlands) was used and it resulted in a pseudo-continuum resembling of sunshine UV. The emitted light from the bulb was collimated by a parabolic mirror and passed a metal net and three stacked liquid filters with quartz windows. The extinction could be varied by changing the filter thickness or the concentration of the liquids. After the light had passed an optional quartz diffuser plate it illuminated homogeneously an area of about 15 cm in diameter. Petri dishes containing the embryos in 20 ml egg water were irradiated. Dishes were examined daily to follow the developmental progress, hatchability, mortality, and malformation. Dead embryos were removed, and embryo media was replaced and kept 20 ml all the time. Spectral compositions of the emitted light could be regulated by varying the thickness and/or concentration of three liquid filters [28]. The calibration of the simulator was accomplished in steps of 1 nm with a spectral radiometer (IS Spectra 320D, Instrument Systems, Germany). After the shell engineering 50 enclosed embryos in one sterile petri dish were exposed under sunshine simulator, and the UV radiation power was 4.70 µW/cm^2^. The embryos were kept in 20 ml beaker at an exposure/dark cycle of 14 h/10 h and they were detected by a fluorescent microscopy (Nikon ECLIPSE TE2400-S, Japan). All the experiments were performed at 28.5°C.

## Supporting Information

Text S1 LbL and biomimetic mineralization.
(0.03 MB DOC)

Text S2 Hatchability.
(0.03 MB DOC)

Text S3 Development.
(0.03 MB DOC)

Text S4 Assessment criteria.
(0.03 MB DOC)

Figure S1 Shell characterization.
(1.20 MB DOC)

Figure S2 Shell spectum.
(0.04 MB DOC)

Figure S3 Viabilities of the embryos.
(1.18 MB DOC)

Table S1 Solutions for LnPO_4_ shell preparation.
(0.03 MB DOC)

Table S2 Intensities and time periods of UVB radiation in Bio-Sun system.
(0.03 MB DOC)
