# Supplementary material for: Guarding Embryo Development of Zebrafish by Shell Engineering: A Strategy to Shield Life from Ozone Depletion
Source: PLoS One. 2010 Apr 1;5(4):e9963. doi: 10.1371/journal.pone.0009963 (PMC2848599; doi:10.1371/journal.pone.0009963)
Supplement: Text S1 — LbL and biomimetic mineralization. (0.03 MB DOC) [file pone.0009963.s001.doc]

**Text S1: LbL and biomimetic mineralization**

All the chemicals used in the experiments were analytic grade. Typical adsorption conditions used to prepare the polyelectrolyte layers on the egg chorions were 0.5 g L^-1^ of chitosan and poly(acrylic sodium) (PAA). These two chemicals have the opposite charges as the requirement of the technique. However, PAA could not deposit onto the yolk-sac of eggs directly since both are negatively charged in solution. Chitosan, a polysaccharide with positive charge, was used to first deposit on the yolk-sac member and then PAA polyelectroytes were deposited by using LbL (Decher 1997). The polyelectrolyte multilayers were built up by alternating the deposition of positive (chitosan) and negative (PAA) layers onto the yolk-sac. The adsorption time of each step was 20 min and embryo concentration was 500 individuals per 50 ml. After each adsorption, the embryos were washed using 0.15 M NaCl to get rid of the residues. The treatments were performed at 28.5ºC.

We took a specific toxicity test to examine the influence of La^3+^, Ce^3+^ and Tb^3+^, concentrations on the embroys. Eggs were collected within 1 h of spawning, rinsed, and placed into a clean petri dish. In Blastula period, the natural eggs of zebrafish were suspended in the different concentration of La^3+^, Ce^3+^, Tb^3+^ for 1 hour. The concentration points were 0.01, 0.05, 0.10, 0.50, 1.00,5.00, 10.00, and 50.00 mM. Then, eggs were collected again and put into 50 ml embryo medium. After 4 days, the viabilities of the eggs were estimated by the larvae amounts. To analyze dose–responses, the concentration causing 50% mortality (LD_50_) was calculated with logistic regression (Logit model). Three parallel experiments were run for each chemical. Results were presented as means ± SD, and probability values of P < 0.05 were considered to be significant.

Decher, G (1997) Fuzzy nanoassemblies: Toward layered polymeric multicomposites. Science 277: 1232-1237.
