# Supplementary material for: Guarding Embryo Development of Zebrafish by Shell Engineering: A Strategy to Shield Life from Ozone Depletion
Source: PLoS One. 2010 Apr 1;5(4):e9963. doi: 10.1371/journal.pone.0009963 (PMC2848599; doi:10.1371/journal.pone.0009963)
Supplement: Text S2 — Hatchability. (0.03 MB DOC) [file pone.0009963.s002.doc]

**Text S2: Assessment criteria**

Hatchability and malformation were used to evaluate UVB effect. Hatchability was calculated as the number of embryos hatched within 4 d after fertilization divided by the total number of embryos. Cessation of heartbeat and circulation were used as end points for mortality. Classification of malformations was based on comparison to control groups using criteria for gross changes in zebrafish development (Kimmel CB *et al,* 1995; Hill AJ *et al,* 2005). Larvae designated as being malformed typically had twisting or kinked spinal deformities and enlarged pericardial sacs. (Dong QX *et al,* 2007). Percent malformation was calculated as the number of embryos having any deformities after hatch divided by the total number of embryos surviving at 4 d. For groups with 100% mortality before hatch, percent of malformation was considered to be 100%. Dishes were examined daily for developmental progress, hatchability, mortality, and malformation. Dead embryos were removed, and embryo media was replaced.

Kimmel CB, Ballard WW, Kimmel SR, Ullmann B and Schilling TF (1995) Stages of embryonic development of the zebrafish. Dev. Dyn. 203: 253-310.

Hill AJ, Teraoka H, Heideman W and Peterson RE (2005) Zebrafish as a model vertebrate for investigating chemical toxicity, Toxicol. Sci. 86: 6-19.

Dong QX, Svobodab K, Tierschc TR and Monroea WT (2007) Photobiological effects of UVA and UVB light in zebrafish embryos: Evidence for a competent photorepair system. J. Photochem. Photobiol. B. 88: 137-146.
