# Supplementary material for: Guarding Embryo Development of Zebrafish by Shell Engineering: A Strategy to Shield Life from Ozone Depletion
Source: PLoS One. 2010 Apr 1;5(4):e9963. doi: 10.1371/journal.pone.0009963 (PMC2848599; doi:10.1371/journal.pone.0009963)
Supplement: Text S3 — Development. (0.03 MB DOC) [file pone.0009963.s003.doc]

**Text S3: Hatchability**

In short term exposure, the specimens in petri dishes (diameter of 9 cm) received UVB irradiation in Bio-Sun system (Vilber Lourmat, Marne-la-Vallée, France) using a radiation wavelength 312 nm. The doses were set in the range of 0.025-0.8 J cm^−2^. The radiation was emitted from a 30-W fluorescent tube (T-20M) above the sample tray and was automatically adjusted 4 times per second by a microprocessor with an error control of less than 1 μJ cm^−2^. Table S2 listed the automatic records for the intensity (2.0-2.61 mW cm^−2^, Table S2) and the time period for the radiation. The irradiation cycles were reproducible, regardless of intensity fluctuation of the UVB source. After mineralization treatment, 50 embryos (in the gastrula stage) in a sterile petri dish were exposed to a preset dose of UVB radiation 0.025, 0.075, 0.125, 0.25, 0.5, and 0.8 J/cm^2^ (within 6 min) in 5 ml egg water in the Bio-Sun system successively (the distance between UVB source and embryos was 10 mm.) and then they were transferred into 20 ml egg water. During the incubation, the lighting cycle was 14 h light and 10 h dark (without UVB). After 3 days, the surviving (hatching successfully) larvae were counted. At least ten independent experiments were performed to investigate the hatchability. The bare embryos were cultured under the same conditions. The experiments were carried out at temperature of 28.5°C. All analyses were performed using SPSS 16.0.

Preparation of egg water. Stock salts: 40 g "Instant Ocean" Sea Salts (Aquarium Systems, Mentor, OH, USA) added to 1 L distilled water. Egg water = 1.5 ml stock salts was added to 1 L distilled water = 60 μg/ml final concentration (Westerfield 2000).

Westerfield, M (2000) The Zebrafish Book: A Guide for the Laboratory Use of Zebrafish (*Danio rerio*). Oregon: University of Oregon Press, 8 p.
