# Supplementary material for: Guarding Embryo Development of Zebrafish by Shell Engineering: A Strategy to Shield Life from Ozone Depletion
Source: PLoS One. 2010 Apr 1;5(4):e9963. doi: 10.1371/journal.pone.0009963 (PMC2848599; doi:10.1371/journal.pone.0009963)
Supplement: Text S4 — Assessment criteria. (0.03 MB DOC) [file pone.0009963.s004.doc]

**Text S4: Development**

Outdoor radiation conditions (long-term exposure) were simulated with a sunshine simulator. A 400-W discharging lamp (MSR 400 HR, Philips, Netherlands) was used and it resulted in a pseudo-continuum resembling UV sunshine. The emitted light from the bulb was collimated by a parabolic mirror and passed a metal net and three stacked liquid filters with quartz windows. The extinction could be varied by changing the filter thickness or the concentration of the liquids. After the light had passed an optional quartz diffuser plate it illuminated homogeneously an area of about 15 cm in diameter. Petri dishes containing the embryos in 20 ml egg water were irradiated. The distance between UVB source and embryos was set to produce a power what we want, 57 mm in our experiment. Spectral compositions of the emitted light could be regulated by varying the thickness and/or concentration of three liquid filters (Dethlefsen *et al,* 2001). The calibration of the simulator was accomplished in steps of 1 nm with a spectral radiometer (IS Spectra 320D, Instrument Systems, Germany). After the shell engineering 50 enclosed embryos in one sterile petri dish were exposed under sunshine simulator to give the UV radiation power of 4.70 µW/cm^2^. The embryos were kept in 20 ml beaker at an exposure/dark cycle of 14 h/10 h and they were detected by using a fluorescent microscopy (Nikon ECLIPSE TE2400-S, Japan). All the experiments were preformed at temperature of 28.5°C.

Dethlefsen V, von Westernhagen H, Tug H, Hansen PD, Dizer H (2001) Influence of solar ultraviolet-B on pelagic fish embryos: osmolality, mortality and viable hatch. Helgol Mar Res 55: 45-55.
