# Supplementary material for: Guarding Embryo Development of Zebrafish by Shell Engineering: A Strategy to Shield Life from Ozone Depletion
Source: PLoS One. 2010 Apr 1;5(4):e9963. doi: 10.1371/journal.pone.0009963 (PMC2848599; doi:10.1371/journal.pone.0009963)
Supplement: Figure S1 — Shell characterization. (1.20 MB DOC) [file pone.0009963.s005.doc]

**A**


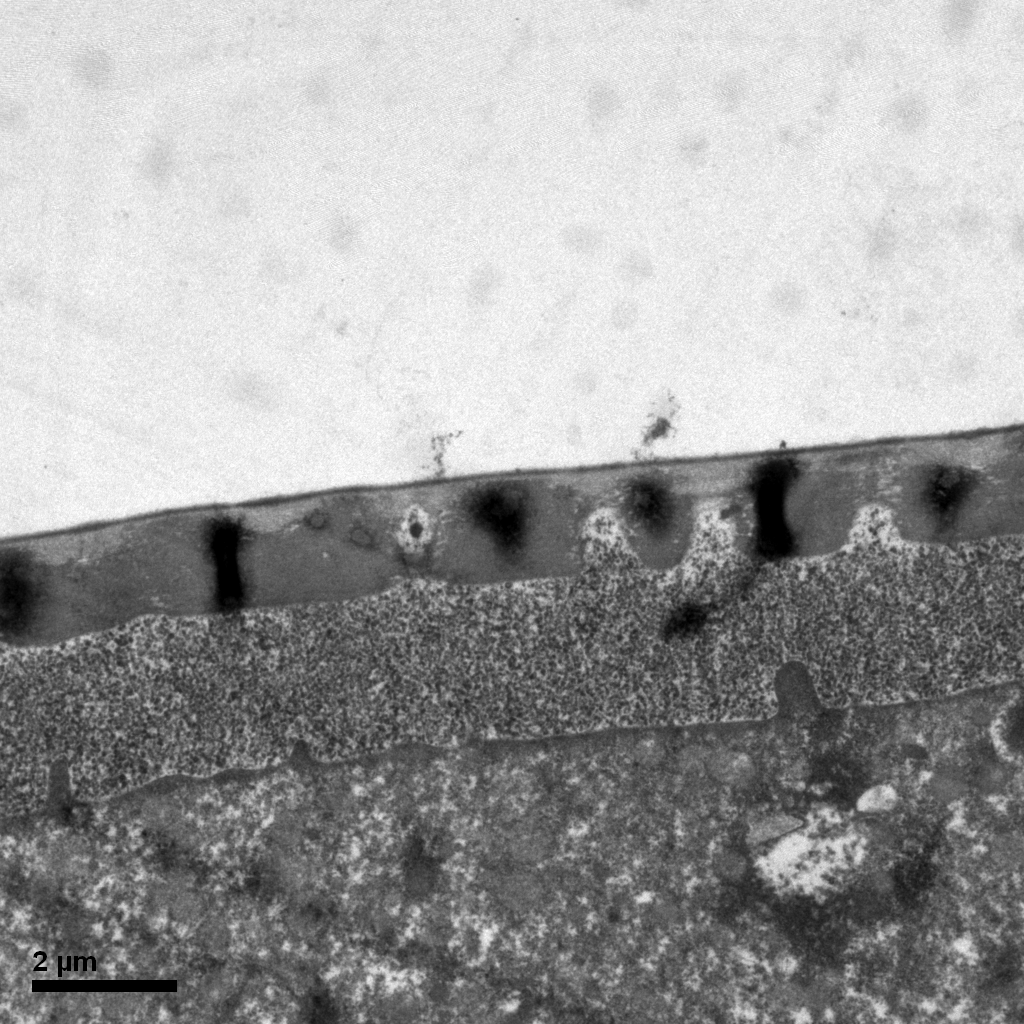


**B**


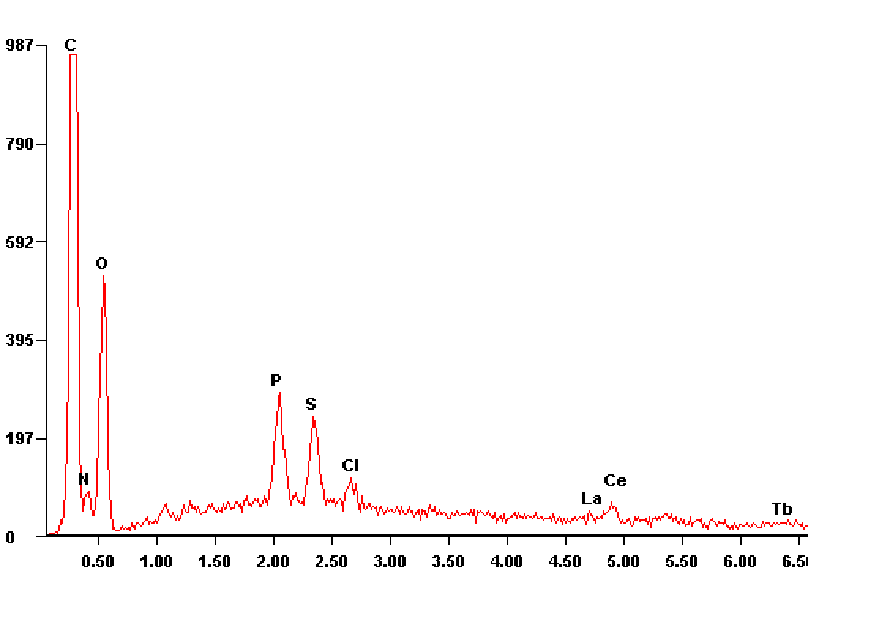
**C**

**Figure S1.** **Shell characterization**

**A.** Transmission electron microscopy (TEM) image of the surface of bare embryos. **B.** The X-Ray Diffraction (XRD) pattern of the mineralized embryos. **C.** The energy-dispersive X-ray spectroscopy (EDX) of the enclosed embryos. La, Ce, and Tb were detected on the surfaces of mineralized embryos. These results confirmed that the embryos were coated by the mineral shell of LnPO_4_. Scale bar, 2 μm.
