# Supplementary material for: Guarding Embryo Development of Zebrafish by Shell Engineering: A Strategy to Shield Life from Ozone Depletion
Source: PLoS One. 2010 Apr 1;5(4):e9963. doi: 10.1371/journal.pone.0009963 (PMC2848599; doi:10.1371/journal.pone.0009963)
Supplement: Figure S2 — Shell spectum. (0.04 MB DOC) [file pone.0009963.s006.doc]

**Figure S2.** **Shell spectum**

Absorption (**A**), excitation (**B**) and emission (**C**) spectra of the LnPO_4_ shell. The broad bands of UVB (280-320 nm) were ascribed to the absorption bands of the cerium in the rhabdophane matrix. The emission spectra, excited by UV rays consisted mainly of four groups of signals, corresponding to the f-f transitions of the Tb^3+^ (450–650 nm), and a weak band (300–400 nm) in the UV region as a result of the d-f transitions of Ce^3+^. The strong emission of Tb^3+^ (547 nm) was due to transitions between the excited ^5^D_4_ state and the ^7^F*_J_* (*J* = 6–3) ground states of Tb^3+^ ions, which was contributed to the green light around the embryos.
