# Supplementary material for: Guarding Embryo Development of Zebrafish by Shell Engineering: A Strategy to Shield Life from Ozone Depletion
Source: PLoS One. 2010 Apr 1;5(4):e9963. doi: 10.1371/journal.pone.0009963 (PMC2848599; doi:10.1371/journal.pone.0009963)
Supplement: Figure S3 — Viabilities of the embryos. (1.18 MB DOC) [file pone.0009963.s007.doc]

**B C**


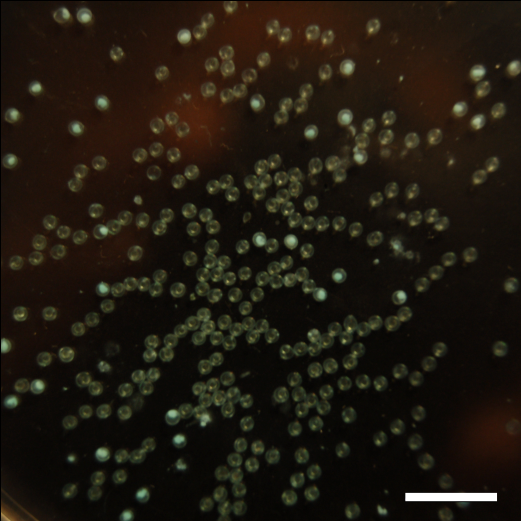

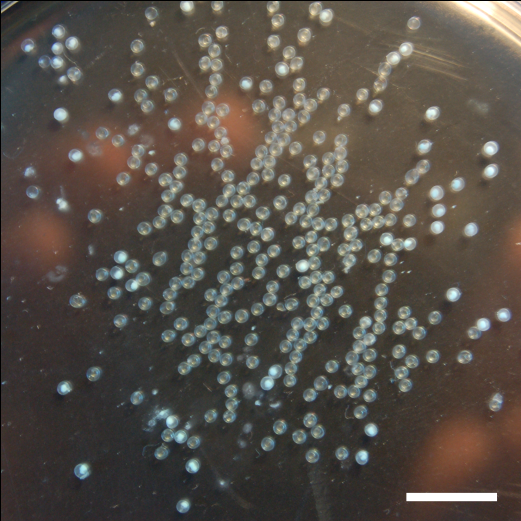


**Figure S3.** **Viabilities of the embryos**

The viabilities of the embryos were recorded statistically during the modification process (A). As shown in Figures S3B and 3C, we can discern the viability of enclosed embryos by their appearances after treatment. The dead embryos were white and their yolks were blurry. However, the viable ones are transparent and bright. We followed up the transparent embryos that we thought alive and the final results showed that almost embryos (~95%) can develop into larvae successfully without abnormal phenotype being detected. During the LbL treatment, 92 ± 6% ones could keep alive (B). After mineralization treatment, 84 ± 7% viability of the eggs could be achieved (C). These values implied the acceptable biocompatibilities of these biomimetic modifications. However, we still suggested that the toxicities could be further reduced by an optimal combination of LbL (by using the other biocompatible polyelectrolytes) and biomimetic mineralization.
