# Supplementary material for: Guarding Embryo Development of Zebrafish by Shell Engineering: A Strategy to Shield Life from Ozone Depletion
Source: PLoS One. 2010 Apr 1;5(4):e9963. doi: 10.1371/journal.pone.0009963 (PMC2848599; doi:10.1371/journal.pone.0009963)
Supplement: Table S1 — Solutions for LnPO4 shell preparation. (0.03 MB DOC) [file pone.0009963.s008.doc]

**Table S1. Solutions for LnPO_4_ shell preparation**

| **Embryo medium** | **1 mM Ln(NO_3_)_3_ solution** | **1 mM Na_2_HPO_4_ solution** |
| --- | --- | --- |
| 0.137 M NaCl  5.4 mM KCl  0.25 mM Na_2_H PO_4_  0.44 mM KH_2_PO_4_  1.3 mM CaCl_2_  1.0 mM MgSO_4_  4.2 mM NaHCO_3_ | 0.137 M NaCl  5.4 mM KCl  0.2 mM Ln(NO_3_)_3_  0.45 mM Ce(NO_3_)_3_  0.35 mM Tb(NO_3_)_3_  4.2 mM NaHCO_3_  Using 1 M NaOH to pH 7.2 | 0.137 M NaCl  5.4 mM KCl  1 mM Na_2_HPO_4_  4.2 mM NaHCO_3_  Using 1 M NaOH to pH 7.2 |
