# Supplementary material for: Guarding Embryo Development of Zebrafish by Shell Engineering: A Strategy to Shield Life from Ozone Depletion
Source: PLoS One. 2010 Apr 1;5(4):e9963. doi: 10.1371/journal.pone.0009963 (PMC2848599; doi:10.1371/journal.pone.0009963)
Supplement: Table S2 — Intensities and time periods of UVB radiation in Bio-Sun system. (0.03 MB DOC) [file pone.0009963.s009.doc]

**Table S2. Intensities and time periods of UVB radiation in Bio-Sun system**

| UVB dose  (J cm^−2^) | Auto-changed intensity (mW cm^−2^) | | Time of radiation (min) |
| --- | --- | --- | --- |
|  | Minimum | Maximum |  |
| 0.025 | 2.00 | 2.39 | 0.19 |
| 0.075 | 2.30 | 2.60 | 0.50 |
| 0.125 | 2.36 | 2.56 | 0.85 |
| 0.25 | 2.29 | 2.59 | 1.27 |
| 0.5 | 2.50 | 2.59 | 3.25 |
| 0.8 | 2.46 | 2.60 | 5.20 |
